# *Trichoderma* Species: Our Best Fungal Allies in the Biocontrol of Plant Diseases—A Review

**DOI:** 10.3390/plants12030432

**Published:** 2023-01-17

**Authors:** Paulina Guzmán-Guzmán, Ajay Kumar, Sergio de los Santos-Villalobos, Fannie I. Parra-Cota, Ma. del Carmen Orozco-Mosqueda, Ayomide Emmanuel Fadiji, Sajjad Hyder, Olubukola Oluranti Babalola, Gustavo Santoyo

**Affiliations:** 1 Instituto de Investigaciones Químico-Biológicas, Universidad Michoacana de San Nicolás de Hidalgo, Morelia 58030, Mexico; 2 Department of Postharvest Science, ARO, Volcani Center, Bet Dagan 50250, Israel; 3 Instituto Tecnológico de Sonora (ITSON), Ciudad Obregón 85000, Mexico; 4 Campo Experimental Norman E. Borlaug, Instituto Nacional de Investigaciones Forestales, Agrícolas y Pecuarias (INIFAP), Ciudad Obregón 85000, Mexico; 5 Departamento de Ingeniería Bioquímica, Tecnológico Nacional de México en Celaya, Celaya 38010, Mexico; 6 Food Security and Safety Focus Area, Faculty of Natural and Agricultural Sciences, North-West University, Private Bag X2046, Mmabatho 2735, South Africa; 7 Department of Botany, Government College Women University Sialkot, Sialkot 51310, Pakistan

**Keywords:** *Trichoderma*, biocontrol agent, bioformulations, mycoparasitism, antibiosis, secondary metabolites

## Abstract

Biocontrol agents (BCA) have been an important tool in agriculture to prevent crop losses due to plant pathogens infections and to increase plant food production globally, diminishing the necessity for chemical pesticides and fertilizers and offering a more sustainable and environmentally friendly option. Fungi from the genus *Trichoderma* are among the most used and studied microorganisms as BCA due to the variety of biocontrol traits, such as parasitism, antibiosis, secondary metabolites (SM) production, and plant defense system induction. Several *Trichoderma* species are well-known mycoparasites. However, some of those species can antagonize other organisms such as nematodes and plant pests, making this fungus a very versatile BCA. *Trichoderma* has been used in agriculture as part of innovative bioformulations, either just *Trichoderma* species or in combination with other plant-beneficial microbes, such as plant growth-promoting bacteria (PGPB). Here, we review the most recent literature regarding the biocontrol studies about six of the most used *Trichoderma* species, *T. atroviride, T. harzianum*, *T. asperellum*, *T. virens*, *T. longibrachiatum*, and *T. viride*, highlighting their biocontrol traits and the use of these fungal genera in Trichoderma-based formulations to control or prevent plant diseases, and their importance as a substitute for chemical pesticides and fertilizers.

## 1. Introduction

The continuing and growing world human population is demanding more food, putting enormous pressure on various agricultural production systems. In this sense, producing more requires more significant extensions of open-field cultivation areas, which are generally dedicated to the cultivation of grains and forages; likewise, greater efficiency and investment in producing fruits and vegetables under greenhouse conditions are needed. To increase plant growth, chemical fertilizers have been used to enhance plant production to the limit of its innate capacities [1].

On the other hand, any agricultural system is exposed to the infection of potential pathogens, be they viruses, bacteria, fungi, or other types of macro-organisms [2], causing serious economic losses each year, which is why, again, the use of chemical pesticides is the first option of many agricultural producers. The main advantage of these pesticides is their immediate use and “solution” to the problem. However, the collateral damage caused by the use of fertilizers and pesticides in the environment and human and animal health has been widely documented [3,4,5,6]. In addition, chemical pesticides induce resistance in pathogens, making them challenging to control after years of continuous application [7]. Fortunately, many countries, mainly in North America and Europe, and some Asian countries, are trying to regulate and decrease its use [8,9,10]. Likewise, the mentality of consumers is changing to organic forms of production, leaving aside large fruits and vegetables and excellent aesthetics. Other developing countries are still struggling with these issues [11].

An important part of sustainable agriculture practices is the control or effective management of plant diseases. Fungi belonging to several genera have been widely used as effective biocontrol agents against fungal phytopathogens, such as *Alternaria, Penicillium, Pichia, Aspergillus,* and *Trichoderma*, with *Trichoderma* being the most used in the field [12,13]. The fungi *Aspergillus terreus* and *Penicillium citrinum* were able to diminish disease symptoms caused by the pathogen *Sclerotium rolfsii*, inducing salicylic and jasmonic acid accumulation in sunflower plants [13], proving to be effective biocontrol agents. Ten endophytic fungi, which include *Penicillium* sp., *Guignardia mangiferae*, *Hypocrea* sp., *Neurospora* sp., *Eupenicillium javanicum*, *Lasiodiplodia theobromae*, and *Trichoderma* sp., showed inhibition under greenhouse conditions against *Fusarium oxysporum* f.sp. *cucumerinum*, the main causal agent of cucumber stem rot disease [14]. Among 32 fungal isolates from the plant *Brugmansia aurea*, *A. aculeatus* inhibited the growth of *F. solani* and *A. fumigatus*, showing potential as BCA [15].

Fungal BCAs are also effective against other kinds of pests, such as insects and nematodes [16,17,18]. Several *Trichoderma* species have been proven to be effective at controlling pests such as *Tetranychus urticae* and different insects that affect important crops [17]. Arbuscular mycorrhizal fungi (AMF) have been widely studied because of their positive effects on plant growth promotion; nonetheless, they are also effective against phytopathogens, such as *Meloidogyne incognita* and other nematodes [18]. The fungus *Arthrobotrys oligospora*, which forms adhesive structures to capture nematodes, is another potential BCA of phytopathogens [19,20]. This information suggests the versatility of fungal BCA to counteract several types of phytopathogens.

Fungal biocontrol agents can also protect plants against abiotic stresses, such as high temperatures [21,22]. They are also used as plant defense enhancers due to their ability to induce systemic resistance, protecting them against several pathogens, all of which lead to an increase in plant yield. In this regard, fungi also have played important roles in enhancing plant growth and crop production [23]. Fungi that can induce plant growth include species from the genera *Trichoderma, Aspergillus, Fusarium, Penicillium, Piriformospora, Rhizoctonia, Colletotrichum, Gliocladium, Phoma,* and others [24,25]. The fungus *Acremonium* sp. showed plant growth-promoting traits on *Allium tuberosum* plants, increasing root and shoot length, as well as antifungal activity against *Botryiosphaeria dothidea* and *Botrytis cinerea* [26]. The fungi *Alternaria* sp., *Phomopsis* sp., and *Cladosporium* sp. increased the biomass of tobacco plants, showing potential as plant growth-promoting fungi [27]. *T. virens* and *T. atroviride* can promote secondary root system development and biomass production of *Arabidopsis* and tomato plants [28,29], being one of the most used genera as plant growth—promoters.

The damage caused by the use of chemical fertilizers and pesticides and the growing use of biocontrol agents presents the need to steer agricultural production systems toward sustainability and stop using synthetic fertilizers and pesticides as much as possible.

An efficient, low-cost, and eco-friendly alternative is the application of microorganisms that promote plant growth and offer protection against pests and pathogens, such as the fungi of the genus *Trichoderma* [30,31]. The use and application of bioinoculants with *Trichoderma* as an antagonistic agent is one of the most active biological control strategies in various countries. In fact, between 50 and 60% of the global market for biological control agents (BCAs) around the world is based on the content of several *Trichoderma* species [32,33]. The controlling action of these *Trichoderma*-based biopesticides mainly includes fungal and oomycete pathogens, such as *Acremonium cucurbitacearum*, *Alternaria* spp., *Aphanomyces cochlioides, Aspergillus* spp., *Lasiodiplodia theobromae*, *Botrytis cinerea*, *Botrytis* spp., *Collisletotnicios* spp., *Collisletnicios* spp., *Diplodia natalensis*, *Fusarium* spp., *Gaeumannomyces graminis* var. *tritici*, *Lasiodiplodia theobroma*, *Phoma betae*, *Rhizoctonia solani, Rhizopus oryzae*, *Pythium* spp., *Serpula* spp., *Sclerotium* spp., *Verticillium dahliae*, among others [32].

*Trichoderma* comprises several species of filamentous fungi that are common inhabitants of the soil, rhizosphere, and endosphere of plants. These fungi have attracted our attention because they can control the growth and infection of potential pathogens such as fungi or nematodes [17]. In this work, these beneficial aspects of different *Trichoderma* species are reviewed, exhibiting different modes of action that benefit many sustainable agricultural production systems.

## 2. An Overview of the Genus *Trichoderma*

The first description of the fungus *Trichoderma* as a genus was in 1794 by Persoon, while Tulasne and Tulasne suggested the sexual state of a *Hypocrea* species in 1865 [34]. Likewise, in 1932 Weindling was a pioneer in proposing *Trichoderma* as a fungus that “parasites” other fungi with the potential to control them [35]. *Trichoderma* species belong to the Hypocreaceae family. They present filamentous hyphae, with optimal growth temperatures between 25 and 30 °C, and they are widely present in various environments, preferring those where there is a decomposing organic matter [36]. *Trichoderma* conidiophores are abundant and end in phialides, pyramidal in shape, and their branches grow in pairs [37]. Asexual conidia are formed abundantly, elliptical in shape, and hyaline, which then develop from white to yellow, and then green conidia when completely mature [36].

Enormous advances have been made in the taxonomy of *Trichoderma*; however, there are still some issues to be resolved when differentiating species within the genus since the vast majority of *Trichoderma* species are not associated with their sexual state and are therefore handled as monoclonal and mitotic. Recent attempts to improve their classification based on barcode oligonucleotide include online tools such as TrichoKEY [38] and DNA Barcoding markers (TrichoMARK), such as internal transcribed sequences (ITS), tef1, and rpb2 genes, to perform specific BLAST type searches (TrichoBLAST) [39]. Recently, Dou and colleagues [40] proposed a Multilocus Identification System (MIST) online for the identification of *Trichoderma* and *Hypocrea* (anamorphs) species for automated detection of 349 *Trichoderma* possible species, also based on a set of three DNA barcodes. Online websites are https://trichokey.com (accessed on 18 December 2022) and http://mmit.china-cctc.org (accessed on 18 December 2022), respectively.

Genome sequencing techniques allowed for a more in-depth study of the genus *Trichoderma*, with *T. virens*, *T. atroviride*, and *T. reesei* being the first species among the genus to have their genome sequenced. This allows a better understanding of their lifestyle as mycoparasites and the difference between species [41].

Some examples of species include *T. harzianum*, *T. aggressivum*, *T. citrinoviride*, *T. asperellum*, *T. ghanense*, *T. hamatum*, *T. koningii, T. pseudokoningii*, *T. virens*, *T. longibrachiatum*, *T. polysporum*, *T. tomentosum*, *T. atroviride*, *T. gamsii*, *T. koningii*, *Hypocrea jecorina/Trichoderma reesei*, *T. spirale*, *T. viridescens*, *T. viride*, and *T. koningiopsis*, which have been found in different ecosystems, such as soils of forests, gardens, decaying wood, cultivated mushroom compost, cereal grains, from various regions of the world, and in marine environments [42,43,44].

*Trichoderma* spp. has been (mostly) considered as non—pathogenic and opportunistic plant symbionts, which can colonize plant roots, establishing a beneficial interaction with their hosts mediated by *Trichoderma* effector proteins and hormonal crosstalk in exchange for plant-derived sugars [45,46,47,48].

During the *Trichoderma–* plant interaction, the benefits received by the plant are not just an increase in biomass and overall nutrition but also protection against several phytopathogens, either by acting directly over the pathogen as a mycoparasite and competing for nutrients or indirectly by inducing the plant defense system [49,50,51,52]. Several species from this genus have been studied and used in field assays as effective biocontrol agents, such as *T. harzianum*, *T. virens*, *T. atroviride*, *T. asperellum*, *T. hamatum*, *T. gamsii*, *T. viride*, among others [30,51,53,54,55,56]. Some of these species will be reviewed further.

## 3. Mechanisms for Protecting Plants Exerted by *Trichoderma*

**Figure 1:**
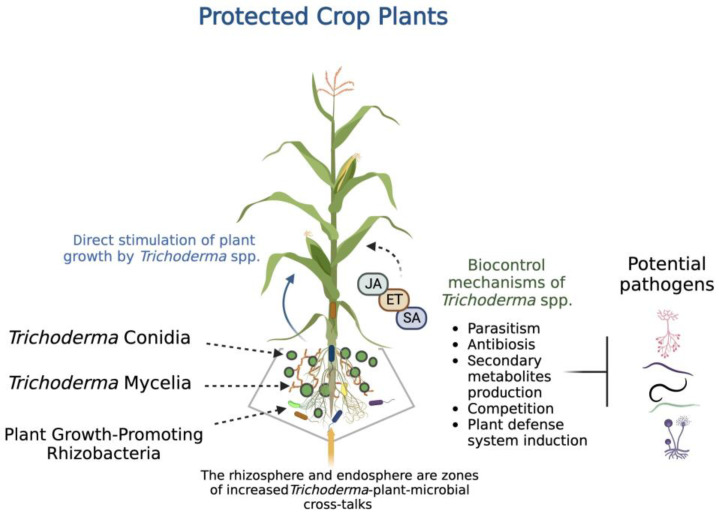
Direct biostimulation and biocontrol properties of *Trichoderma* species. Beneficial *Trichoderma* spp. exert fungal-root communication via diffusible and volatile compounds, regulation of the stress hormone ethylene, and production of phytohormones, such as auxins (indole-3-acetic acid). Some of the plant-protecting mechanisms of *Trichoderma* include parasitism, antibiotic and secondary metabolites production, or activation of the induced systemic resistance (ISR). *Trichoderma* can trigger both growth-stimulating effects and plant defense action by the elicitation of salicylic acid (SA), ethylene (ET), and jasmonic acid (JA) dependent pathways against several types of potential plant pathogens such as nematodes and fungi.

The biocontrol mechanisms exerted by *Trichoderma*, which lead to efficient plant protection, can be direct when the fungus interacts with the pathogen by mycoparasitism, competition, or antibiosis; and indirect if the fungus enhances plant defense systems so the plant can defend itself against its pathogen [57,58]. Additionally, *Trichoderma* spp. can exert diverse direct plant growth-promoting activities by producing some molecules, such as phytohormones. Figure 1 depicts the biocontrol properties of *Trichoderma*, exerting protection on crop plants.

### 3.1. Mycoparasitism

Mycoparasitism is one of the main mechanisms of inhibition of the mycelial growth of fungal pathogens, providing nutrients to the mycoparasite when they kill their prey. In some cases, *Trichoderma* obtains the nutrients but does not kill the pathogen (biotrophic mycoparasites). In work by Kubicek and collaborators [41], they compared the genome of three *Trichoderma* species (*T. reesei*, *T. virens* and *T. atroviride*), as well as their respective teleomorph or sexual forms (*Hypocrea jecorina*, *H. virens* and *H. atroviridis*, respectively). The authors found high conservation of the genetic origin (up to 96%), in addition to the fact that several genes that code for antagonistic or mycoparasitic activities are conserved in these species, suggesting that mycotrophy is an ancestral lifestyle in this genus [41,59].

There are three main steps during the act of mycoparasitism. This function can be carried out in the rhizosphere of plants, an ecosystem efficiently colonized by *Trichoderma* and where the biological control of potential pathogens is important to avoid plant diseases. First, *Trichoderma* requires recognition of the host (or possible plant pathogenic fungus), where the production of oligochitins has been proposed as sensor molecules [60]. Likewise, it is known that during this previous step, various genes that encode proteases and oligopeptide transporters are expressed before contact with the fungal host. Second, hydrophobin-like proteins may have a relevant function once *Trichoderma* encounters the plant-pathogenic fungus, which leads to the formation of papillae or appressoria-like structures. The third step occurs when *Trichoderma* coils around the pathogen hyphae and starts degrading it via the production of cell-wall degrading enzymes, such as cellulases and hemicellulases, chitinases, proteases, and -1,3-glucanases, among other secondary metabolites, that are essential for mycoparasitism [60]. It should be noted that the host that is being parasitized also produces metabolites and reactive oxygen species (ROS) as defense mechanisms in response to the attack, which in turn, *Trichoderma* turns on genes involved in detoxification and response to stress. Interestingly, these lytic proteins are also produced and purified using *Trichoderma* as a host for biotechnological purposes [61].

### 3.2. Antibiosis

The biological control mechanism known as antibiosis involves the production and excretion of secondary metabolites, which include compounds of a different chemical nature with cytotoxic activity, that can limit or inhibit pathogen growth. Antibiosis is one of the main modes of action of *Trichoderma* and other biological control agents, such as plant growth-promoting bacteria (PGPB) [34,51,62]. In fact, the expression of coding genes to produce antibiotic metabolites is increased in the presence of pathogens and compounds produced by plants, exerting a stimulating effect of protection and fine signaling between the plant, the pathogen, and the biocontrol agent [34,51].

**Figure 2:**
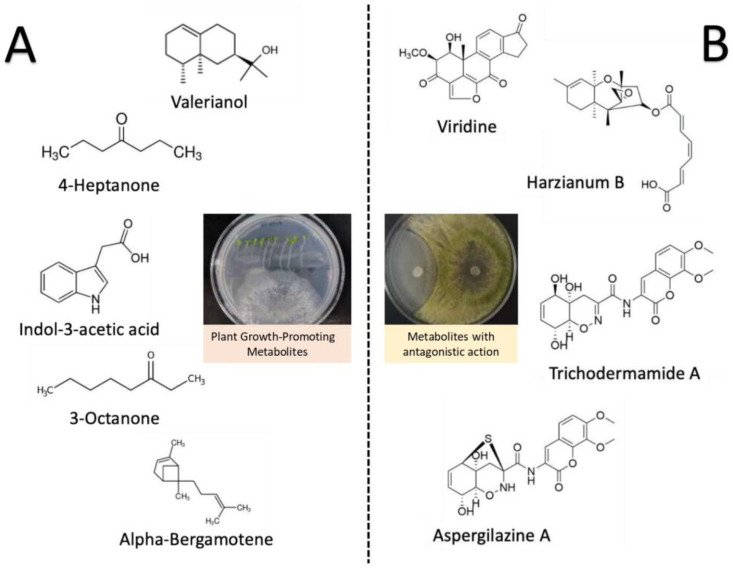
Examples of *Trichoderma* secondary metabolites involved in plant interactions with growth-promoting effects (**A**) and secondary metabolites involved in mycoparasitism with antibiotic effect (**B**).

The various species of *Trichoderma* are a factory of secondary metabolites, as more than 180 different types of compounds have been proposed and can be classified according to their function in competition and iron-quelating metabolites, inducers of plant resistance, plant growth-promoting metabolites, antibiotics, and if the metabolites are volatile or non-volatile [63,64,65]. For example, *T. virens* species produce trichodermamides, while *T. koningii* synthesizes Koninginins, both with antimicrobial and antifungal activity [66,67]. Furthermore, in *T. harzianum* and *T. virens,* compounds such as azaphilones, viridins, nitrogen heterocyclic compounds (e.g., harzianopyridone and harzianic acid), and volatile terpenes have been characterized, and are involved in the biocontrol of pathogenic fungi [30]. The production of hydrolytic enzymes and proteases, such as exo- and endochitinases, chitinases, xylanases, glucanases, lipases, endo-and exopeptidases, among others with antifungal action, have also been characterized in different *Trichoderma* spp. [68]. The volatile organic compound (VOC) 6-pentyl-2H-pyran-2-one (6-pentyl-α-pyrone, 6-PP) is the most abundant VOC from *T. atroviride*, and it enhances plant growth and regulates sugar transport in *Arabidopsis* roots, along with other VOCs produced by the fungus [69]. Figure 2 shows a glimpse of the metabolite’s arsenal involved in fungal antagonism and some compounds involved in plant growth promotion (e.g., indol-3-acetic acid).

### 3.3. Competition

Bulk and rhizospheric soil are complex ecosystems with a continuous battle to access resources and maintain survival. In the rhizosphere, a much richer environment than bulk soil due to the excretion of nutrients by the plant roots, such as amino acids, vitamins, organic acids, saccharides, etc., competition is an essential strategy for survival [70,71]. For this reason, those organisms residing in the rhizosphere with efficient metabolic and competitive capacities will access the best “sites” where the resources exist. In this sense, Trichoderma species, as mentioned before, are capable of producing a series of antagonistic compounds (e.g., antibiotics or lytic enzymes), which, in conjunction with further rapid growth and colonization strategies (e.g., metabolic versatility), they can occupy spaces in the rhizosphere and, directly, benefit the growth of plants and restricting the development of other potentially pathogenic microorganisms [72,73]. However, this strategy is also employed by PGPB, which exhibits efficient colonization mechanisms to occupy rhizospheric spaces and endophytic regions [74]. Therefore, when selecting Trichoderma biocontrol species (or other biocontrol microorganisms), it is important to perform antagonism tests towards beneficial organisms for plants, such as PGPB [75] to determine their synergistically or detrimental potential among each other.

### 3.4. Induction of Plant Defense System

When attacked by various pathogens or mechanical damage, plants turn on defense systems that allow them to protect themselves, such as systemic acquired resistance (SAR) [76]. In some other cases, plant-associated microbes can induce the plant defense systems, such as the rhizobacteria-induced systemic resistance (RISR) pathway, which phenotypically resembles SAR [1], in response to the presence of the microorganism. It could be said that *Trichoderma*-induced systemic resistance (TISR) is very similar to RISR since both are regulated by the jasmonic acid and ethylene (JA/ET) signaling pathway [52,77,78]. However, the plant defense system and the signaling that coordinates the response are highly variable, even within the same plant kingdom. In fact, Bakker et al. [78] mention that in RISR, there is no induction of the expression of pathogenesis-related proteins (PR), as in SAR, which is stimulated by the attack of fungal or herbivore pathogens. Nonetheless, *T. hamatum* strain Th23 can induce PR-1 and PR-7 expression in tomato plants upon infection with Tobacco Mosaic Virus (TMV)[79]. The overall plant response to pathogens includes the production of antifungal glucanases and chitinases, thaumatins, as well as the synthesis of oxidative enzymes, including peroxidases (POD), polyphenol oxidases (PPO), and lipoxygenases [80] and the activation of several transcription factors involved in the plant immune response to biotic stressors [81]. *T. hamatum* strain Th23 induces CAT, SOD, and PPO enzymatic activity in tomato plants during infection with TMV and reduces H2O2 and malondialdehyde (MDA) concentrations [79]. One of them is NPR1, a transcription factor that is widely known for its action in modulating both SAR and RISR pathways [82]. *T. harzianum* TR 274 induces the expression of several defense-related genes in *Phaseolus vulgaris* plants, such as glu, chit, and pal [83], which are genes related to TISR; and the commercial formulation BIOSPARK™, made from *Trichoderma* spp. induces resistance in *Lansium domesticum* plants against the insect *Unaspis mabilis* [84]. Some TISR elicitor compounds are homologous to those produced by rhizobacteria, such as siderophores, acyl-homoserine lactones, and antimicrobial compounds, among others [85,86]. It should be taken into account that the TISR response has been little studied compared to RISR. *Trichoderma* elicitors may be regulated in different ways according to the species; for example, SM1/EPL1 from *T. virens* and *T. atroviride* induce a defense response in plants, but SM1 from *T. harzianum* seems to be downregulating plant defense responses, allowing root colonization [83]. This suggests that species and type of elicitor are important factors to consider when inducing TISR in plants, so it is necessary to delve further into the elicitors and induction pathways of plant defense systems.

## 4. Biocontrol Potential of Registered *Trichoderma* Species

With over 200 *Trichoderma* species registered [40,87] and their potential to be used as biocontrol agents and plant growth promoters, it has led to many studies to gain more knowledge about their mechanisms of action, focusing studies on mycoparasitism and competition [60,88], production of secondary metabolites with antagonistic activity [63,68] and induction of plant systemic resistance [52,89].

Among the registered *Trichoderma* species, *T. harzianum*, *T. asperellum*, *T. atroviride*, *T. longibrachiatum*, *T. viride*, and *T. virens* are the most sampled ones [55]. The first three species are among the most used biocontrol agents, using mycoparasitism and competition as their primary mechanism of action against fungal phytopathogens. Meanwhile, species such as *T. virens*, *T. longibrachiatum*, and *T. viride* use antibiosis as a strong mechanism of action against several plant pathogens [50,88,90,91]. Here, we review the *Trichoderma* species mentioned above, focusing on their biocontrol traits and the most recent literature on this subject, summarized in Table 1.

### 4.1. Trichoderma Atroviride

*Trichoderma atroviride* is a filamentous fungus that can be isolated from soil, mainly in temperate climates, with optimal growth at 25 °C, presenting thin and hyaline colonies and inconspicuous aerial hyphae and gray to dark green conidia after 2 to 7 days [173]. It has a characteristic coconut smell due to the production of the volatile compound 6-pentyl-2H-pyran-2-one, or 6-PP, which also is involved in biocontrol against several plant pathogens, including *Cylindrocarpon destructans, Mcrophomina phaseolina*, *Phytophthora* sp., and others, and it is also involved in plant growth-promotion and induction of systemic resistance [174,175,176].

*T. atroviride* has biocontrol capacity on different plant pathogens, including fungi, oomycetes, and pests, such as nematodes or insects, by exerting different mechanisms of biocontrol [17,31], which are presented below with recent examples from the literature.

#### 4.1.1. Parasitism and Competition

In a competition for space and nutrients, *T*. *atroviride* inhibits *Phytophthora cinnamomic* growth and zoospore formation. In a tripartite interaction with tomato plants, *T. atroviride* enhances protection against the disease induced by this oomycete [93]. Also, *T. atroviride* competes with and is capable of antagonizing *Fusarium avenaceum* and *Fusarium culmorum*, important maize pathogens [95], and the grapevine pathogen *Neofusicoccum parvum* [94]. In dual culture assays, *T. atroviride* can parasitize several fungal pathogens, including *Neofusicoccum batangarum*, *N. parvum*, *Phytophthora nicotianae*, *Penicillium digitatum*, *P. commune*, *P. roqueforti*, *P. verrucosum*, *Aspergillus steynii*, *Fusarium proliferatum*, *F. verticilloides*, *F. sporotrichoides* and *F. poae* [92].

As stated before, the main mechanism used by *T. atroviride* as a biocontrol agent against fungal pathogens is mycoparasitism [50]. Nonetheless, this fungus also uses other strategies to limit the growth of different plant pathogens.

#### 4.1.2. Secondary Metabolites

The effect of secondary metabolites as a biocontrol trait can be tested as whole fungal cultures or extracts, as individual components, or as a mix of components that had been identified from the whole extract. Whole fungal culture (soluble metabolites) from a local *T. atroviride* strain BC0584 and its volatile organic compounds (VOCs) are capable of inhibiting the growth of *F. avenaceum*. In contrast, both soluble metabolites and VOCs did not have any statistical significance in inhibiting the growth of *F. culmorum.* Nonetheless, in confrontation assays, both pathogens are controlled by *T. atroviride* BC0584 [95]. The production of VOCs is a characteristic of *Trichoderma* species, and 6-PP is probably the most characterized VOC from the species that synthesize this compound, such as *T. atroviride* [175]. The synthesis of 6-PP is regulated by dark conditions when it is produced in more quantities. It enhances the antagonistic activity of *T. atroviride* P1 and IMI 206,040 strains against *R. solani* and *F. oxysporum* [100]. Fermented cultures are used to obtain certain metabolites, such as antibiotics, and are obtained at the end of several days of the fungus growing in a liquid medium [177]. The fermented culture from *T. atroviride* CCTCCSBW0199 could inhibit the growth of *B. cinerea* in an in vitro assay to 73% [98], indicating an antibiosis mechanism to control the pathogen.

In a broad-range pathogen study, Stracquadanio and collaborators [92] found that the ethyl acetate extract and the fungal filtrate from *T. atroviride* (TS) inhibit growth and have strong cytotoxic activity against 25 pathogens, which includes 7 species of *Penicillium*, 6 species of *Aspergillus*, 6 species of *Fusarium*, 2 species of *Neofusicoccum*, 2 species of *Colletotrichum*, and 2 species of *Phytophthora*. The velvet complex proteins in *Trichoderma* are involved in several physiological processes, including secondary metabolite synthesis [91]. In a study to unravel the role of *vel1*, a member of the velvet complex in *T. atroviride* T23, Karuppiah and collaborators [97,143] found that the fungal extract of the wild-type strain and the *vel1* overexpressing strain, both alone and in the co-culture with *Bacillus amyloliquefaciens* 1841, inhibit the growth of the wheat pathogen *F. graminearum*, and decrease the disease severity in plants treated with those strains; the authors also note that the co-cultures have better inhibition rate over the pathogen, and induce a stronger plant resistance that the single cultures [97].

Swollenins are proteins with similarity to plant expansins and are involved in the remodeling of plant cell walls and colonization [178]. TaSWO1, a swollenin secreted by *T. atroviride*, can induce resistance in *Capsicum annum* plants against *A. solani* and *R. solani*, reducing the symptoms caused by these pathogens [96]. The LysM effector identified as Tal6 from *T. atroviride* IMI 206,040 binds fungal chitin, preventing the plant from sensing the BCA, allowing it to establish a beneficial interaction, and enhancing *T. atroviride* mycoparasitic activity against *B. cinerea, Sclerotium cepivorum*, *Colletotrichum lindemutianum* and *R. solani* AG2 [99].

The capacity of *T. atroviride* to produce a wide range of volatile and non-volatile secondary metabolites is indicative of its capacity to control different types of phytopathogens, which makes this fungus a capable BCA in many agricultural situations.

#### 4.1.3. Plant Defense Induction and Priming

*Trichoderma*, colonization of roots, can induce different plant defense responses; for example, *T. atroviride* SC1 induces SA-mediated defense response in grapevine Tempranillo cultivar, enhancing the plant protection against *N. parvum*, which is also inhibited in dual cultures with *T. atroviride* [94]. Leal and collaborators (2021) also performed co-cultures of *T. atroviride* with *B. subtilis* PTA-271. They found that co-culture is better at enhancing plant protection than the inoculation of single organisms.

Some of the secondary metabolites produced by *T. atroviride* can induce plant resistance; for instance, fermented culture from *T. atroviride* CCTCCSBW0199 alone or in combination with brassinolide increases peroxidase (POD) and superoxide dismutase (SOD) activity in tomato plants, which then increases the plant resistance and reduces the symptoms induced by *B. cinerea* [98].

*Trichoderma* species, when colonizing the plant, alter plant transcriptome, modifying gene transcription involved in plant defense responses, such as *T. atroviride* P1, which modifies gene transcripts related to plant defense, and induces plant-defense related VOCs to attract aphid-predatory wasps *Aphidius ervi*. They provide a better defense mechanism against the aphid *Macrosiphum euphorbiae* and the moth *Spodoptera littotalis* in tomato plants [102], proving that *T. atroviride* may be able to control pathogens indirectly, modulating plant physiology. Besides modification of the transcription of genes involved in plant defense responses, colonization by *T. atroviride* also induces *Arabidopsis*’s sRNA-mediated gene silencing, leading to an increase in gene expression of JA and SA-mediated pathways, which in turn induces priming and increases resistance in the plant against *B. cinerea* [103].

Plant defense induction by *T. atroviride* is an important mechanism of action for this BCA due to the span of plant pathogens that can be controlled with it since direct mechanisms such as mycoparasitism may be limited. Still, it can be complemented with indirect mechanisms. Therefore, *T. atroviride* is an effective antagonist of several fungal, oomycete, insect, and other plant pathogens.

### 4.2. Trichoderma Harzianum

*T. harzianum* is frequently found in temperate climates, with optimal growth at 30 °C, but can grow fine at 35 °C; conidiation is presented at day 2 in concentric zones when growing in Petri dishes, changing from green to dark green/brownish in color; unlike *T. atroviride*, *T. harzianum* has no particular odor [37,179].

This fungus is found in several substrates such as soil, other fungi, decaying plant material, and as an endophyte of several plants, acting as a biocontrol agent for different soil-borne diseases. It has been used widely in agriculture, being one of the active ingredients of commercial products used to control crop diseases and to promote plant growth and yield [179].

As a biocontrol agent, *T. harzianum* is efficient at inhibiting plant pathogens such as *Fusarium solani* or mycotoxin—producing fungi by competition, antibiosis, and inducing plant defense responses [109,180]. It is also a biocontrol agent of pests such as aphids by inducing the plant defense system against them [126]. Examples of direct and indirect mechanisms of biocontrol from *T. harzianum* are presented below.

#### 4.2.1. Parasitism and Competition

In an in vitro assay of *F. oxysporum* f.sp. *lycopersici* in confrontation with five *Trichoderma* species, all the species were able to inhibit the pathogen’s growth, being both *T. harzianum* strains tested, BHU-BOT-RYRL4 and MTCC936, the ones that inhibited the pathogen’s growth the most (83.17% and 72.13%, respectively) [104]. In a rhizosphere colonization assay with wheat plants, *T. harzianum* Tr904, as well as *T. gamsii* and *T. afroharzianum*, colonized the rhizosphere and competed for space and nutrients with the pathogen *Fusarium pseudograminearum*, preventing plant disease caused by this pathogen [110].

In the dual confrontation of *T. harzianum* against *Fusarium sudanense*, the BCA parasites the pathogen degrading its hyphae and inhibiting its growth by also competing for space and nutrients, preventing seed rot in wheat plants [105]. *T. harzianum* has also shown antagonistic ability in vitro during the confrontation with the pathogen *Alternaria cerealis*, limiting its growth [106], and the strain *T. harzianum* T-soybean showed mycoparasitic activity against *F. oxysporum,* reducing its growth by 45.45% [107].

In dual confrontation assays, two strains of *T. harzianum*, CMML20-26 and CMML20-27, showed strong antagonistic activity against several sweet potato postharvest pathogens, including *Fusarium ipomeae, F. oxysporum, F. solani*, *Penicillium citrinum¸ P. rotoruae, Aspergillus wentii, Mucor variicolumellatus* and *M. phaseolina* [108]. *T. harzianum* MRI001 can mycoparasite *F. oxysporum*, *A. alternata, Aspergillus carbonarius,* and *A. flavus*, overgrowing the pathogens and reducing the production of the mycotoxins ochratoxin and aflatoxin B_1_, produced by *A. carbonarius* and *A. flavus* respectively [109]. In a confrontation assay, *T. harzianum* inhibits the growth of the chili pepper pathogen *Colletotrichum truncatum*, competing for space [112]. Several Egyptian *T. harzianum* strains showed mycoparasitic activity against *F. graminearum*, *M. phaseolina*, and *F. solani*, the strain *T. harzianum Th6*, the most effective one against all three pathogens [111,181].

*T. harzianum* has proved to be an effective mycoparasite, not only by degrading its host hyphae but by competing for space and nutrients against a wide range of fungal plant pathogens. These characteristics make this fungus a competent BCA against soil-borne fungal pathogens, with prominent applications in agriculture.

#### 4.2.2. Secondary Metabolites

*T. harzianum* is also a good producer of secondary metabolites with important biocontrol traits. Inoculation of *T. harzianum* induces VOCs production in maize roots, and the exogenous application of 6-PP diminishes the damage caused by the root herbivore *Phyllophaga vetula* [113], indicating that the volatiles produced by the fungus induces resistance in the plant, even though, no direct biocontrol of *P. vetula* was observed by *T. harzianum*. ThMBF1 is a transcriptional coactivator from *T. harzianum* T34 involved in the synthesis of several secondary metabolites. Its regulation is vital to maintaining biocontrol capability over *B. cinerea* and *F. oxysporum* since its overexpression significantly reduced the BCA capacity to inhibit the pathogens’ growth and its capacity to confer resistance in tomato plants [114].

Some important secondary metabolites from *Trichoderma* species are peptaibols, which are involved in antibiosis activity against several plant pathogens [63]. Three peptaibols from *T. harzianum* HK-61, named trichorzins HA II, HA V, and HA VI, reduced lesions caused by *Cucumber mosaic virus* (CMV) up to a 90% (trichorzin HA V) in *Vigna sesquipedalis* plants [115].

Other important secondary metabolites are proteases. The aspartic protease P6281 from *T. harzianum* GIM 3.442 significantly reduces the growth of *B. cinerea, Mucor circinelloides, A. flavus, A. fumigatus,* and *R. solani*, disrupting the cell wall integrity of *B. cinerea*, preventing it from causing lesions in fruits such as orange and apples, and inhibiting spore formation in *B. cinerea*, *M. circinelloides*, *A. flavus*, and *A. fumigatus* [116].

Secondary metabolites can have antibiosis or antimicrobial activity. SM present in the fungal extract of *T. harzianum* CCTCC-RW0024 strain showed antifungal activity against *F. gramineraum,* inhibiting the pathogen growth up to 96.3% and conferring resistance in maize plants [73]. The culture filtrate from *T. harzianum* T-soybean has antifungal activity against *F. oxysporum,* inhibiting the pathogen growth up to 60.4%, granting resistance in soybean against *F. oxysporum* [107]. Culture filtrate from *T. harzianum* can inhibit the growth of the bean pathogen *Pythium ultimum* and, combined with chamomile extract, reduces disease symptoms in *Phaseolus vulgaris* seeds caused by this pathogen [120]. Cell-free culture filtrates improved in chitinase activity, showed fungal growth inhibition against the pathogens *Dematophora necatrix*, *F. solani*, *F. oxysporum*, and *Pythium aphanidermatum*, and the effect is concentration-dependent, were at a concentration of 25% of the filtrate showed the maximum growth inhibition rate for all the pathogens tested [121]. The G-protein signal regulatory mechanism is involved in fungal processes such as pathogenesis and secondary metabolism synthesis [182], and the Thga3 subunit from *T. harzianum* Th33 is involved in its mycoparasitic ability against *R. solani*, regulating chitinase activity, hydrophobicity, and growth of the fungus [117].

Some secondary metabolites produced by *T. harzianum* can induce plant defense mechanisms, such as the flavoenzyme ThLAAO (L-amino acid oxidase) from *T. harzianum* ETS323, which has antibiotic activity, and when it is expressed in tobacco plants, induces the expression of genes related to SA-, JA- and Et- mediated defense pathways, as well as ROS accumulation, conferring resistance to *Sclerotinia sclerotiorum* and *B. cinerea*, and resistance to *B. cinerea* in cabbage plants [118]. The expression of the gene *thkel1* from *T. harzianum* CECT 2431 in *Arabidopsis* and *Brassica napus* plants caused resistance to *B. cinerea* in *Arabidopsis* and *P. lingam* in rapeseed, increasing the expression of genes related to SA- and JA- mediated defense pathways [123]. Other SM can regulate virulence genes from the pathogen, such as Epl-1 from *T. harzianum*, which represses virulence genes in *B. cinerea*, and induces SA- mediated defense pathway and priming in tomato plants, conferring resistance to the pathogen [119]. SM from culture filtrates of *T. harzianum* and the fungus diminished the adverse effects that *Fusarium culmorum* causes in wheat plants, such as reduced germination or lower plant growth, and modified antioxidant enzymatic activity, overall conferring protection against the pathogen [122].

*T. harzianum* is a proficient secondary metabolites producer. This ability works in its favor as a BCA, regulating and inhibiting the growth of several phytopathogens and using its SM to induce plant resistance, protecting plants not just in a direct manner but indirectly as well. This makes the study of secondary metabolites produced by *Trichoderma* an important subject to take advantage of in agriculture.

#### 4.2.3. Plant Defense Induction and Priming

*T. harzianum* is effective at inducing plant defense systems against insects, such as with the feeding insect *Nezara viridula*, whose growth is impaired in tomato plants that had been inoculated with *T. harzianum* T22, also inducing the expression of *loxD* and *PIN2* genes, related to the JA-mediated defense pathway in the plants [124]. *T. harzianum* T22 also induces a strong plant VOCs priming in tomatoes, attracting the parasitoid *A. ervi*, so the plants can defend themselves against the aphid *M. euphorbiae* [126], reprogramming the plant transcriptome and metabolome to favor induction of JA, Et and ISR defense pathways, and increasing isoprenoid biosynthesis, leading to a strong defense response against *M. euphorbiae* [127].

Besides, *T. harzianum* can also induce resistance against other pathogens, such as nematodes. *T. harzianum* diminishes infection symptoms caused by the nematode *Meloidogyne incognita* in tomato plants, inducing the gene expression of *PR1*, *PR5*, *JERF3*, and *ACO*, which are related to SA- and JA/Et- mediated defense responses in plants [125].

The production of ROS is one of the defense responses in a plant that can be induced by beneficial microbes, such as *T. harzianum* induction of accumulation of H_2_O_2_ and other important defense-related enzymatic activity, such as SOD, in tomato plants, upon infection with *F. oxysporum* f.sp. *lycopersici* [104]. To confer protection against *F. oxysporum*, *T. harzianum* colonizes cucumber roots reducing ROS and reactive nitrogen species (RNS) accumulation caused by the pathogen, promoting redox homeostasis, and increasing antioxidant enzymatic activity to enhance plant protection [129]. *T. harzianum* induced priming, defense-related enzymatic activity (PAL, POX, PPO), as well as antioxidant enzymatic activity (SOD, catalase (CAT), and others) in chili pepper plants upon infection with *C. truncatum*, also diminishing the symptoms caused by the pathogen and ROS induced accumulation, protecting the plant against its pathogen [112].

A defense-related enzymatic activity such as PAL, POX, CAT, and ascorbate peroxidase is induced by inoculation with *T. harzianum* UBSTH-501 in wheat plants, conferring resistance against *Bipolaris sorokiniana* infection, and promoting SA and phenolic compounds accumulation, as well as lignin and suberization in leaves, to reinforce plant defense [128]. Upon infection of tomato plants with *A. cerealis*, *T. harzianum* induces the accumulation of different phenolic compounds, such as flavonoids and terpenoids, and increases the plant antioxidant enzymatic activity, diminishing the infection caused by *A. cerealis* [106]. Soybean plants treated with *T. harzianum* T-soybean showed less cellular death caused by *F. oxysporum* and increased protection against the pathogen [107].

It is worth noting that the induction of plant defense systems mediated by *T. harzianum* causes the plant to reprogram its metabolite synthesis to favor the production of compounds that can help the plant to defend itself against pathogens that cannot be directly attacked by *T. harzianum*, such as aphids or viruses, conferring this way a whole protection against different kinds of phytopathogens.

### 4.3. Trichoderma Asperellum

*T. asperellum* grows well in temperatures ranging from 25 °C to 30 °C, and it is a cosmopolitan species, found frequently in agricultural and undisturbed soils and plant material, with lifestyles ranging from saprotrophy to biotrophy [183]. Conidia appear after 5 days and are dark green in color, forming at the center of the colony in Petri dishes [135].

Along with *T. atroviride*, *T. asperellum* is considered a strong mycoparasite of different plant pathogenic fungi by competition, hyperparasitism of host or antibiosis, and it can induce plant resistance [112,135,184]. Below, we present examples of the direct and indirect biocontrol mechanisms used by *T. asperellum*.

#### 4.3.1. Parasitism and Competition

In dual culture between several strains of *Trichoderma*, with the phytopathogens *Fusarium camptocerus*, *F. oxysporum*, *A. alternata*, *F. solani*, *Colletotrichum gleosporoides*, *Ganoderma applanatum*, *B. cinerea* and *Cytospora chrysosperma*, the *Trichoderma* strain TaspHu1, identified as *T. asperellum*, showed better biocontrol traits, inhibiting the growth of the pathogens by showing mycoparasitic activity and competition for space and nutrients [130]. In a dual confrontation assay, *T. asperellum* inhibits the growth of the chili pepper pathogen *Colletotrichum truncatum*, competing for space [112].

In dual confrontation assays, *T. asperellum* IMI393899 showed a mycoparasitic capacity of several fungal pathogens, including *Neofusicoccum batangarum*, *N. parvum*, *C. gloeosporoides*, *Phytophthora nicotianae*, *Phytophthora parvispora*, *Penicillium digitatum*, *P. roqueforti*, *P. verrucosum*, *Fusarium proliferatum*, *F. sporotrichoides*, *F. langsethiae*, *F. graminearum* and *F. poae* [92]. *T. asperellum* T1 showed antifungal activity in dual confrontation assays against the pathogens Corynespora cassiicola and Curvularia aeria, the causal agents of leaf spot in lettuce, inhibiting the growth of the pathogens, and overgrowing them in the Petri dish [134].

Confrontation of *T. asperellum* 6S-2 against the apple pathogen *Fusarium proliferatum* f.sp. *malus domestica* MR5 leads to the degradation of the pathogen mycelia during the mycoparasitic interaction [133]. In dual culture antagonism assays, *T. asperellum* TA showed mycoparasitic activity against the white-rot fungus Phellinus noxius, conferring resistance in *Eryobotria japonica* plants in an in planta assay, showing fewer symptoms caused by the pathogen [136]. Additionally, *T. asperellum* GDSF1009 showed biocontrol traits by competing and mycoparasite the pathogens *F. oxysporum* f.sp. *cucumerinum* Owen and *F. graminearum*, reducing their growth [135].

*Trichoderma* species are also used in agriculture because some of them are resistant to several abiotic stresses. *T. asperellum* ACCC30536 salt-tolerant mutants T3 and T5 showed antifungal capacity against *R. solani* and *A. alternata* under salt stress conditions, conferring resistance in PdPap plants by activating SOD, CAT, and POD enzymatic activities [132]. This shows the potential of isolating strains from extreme environments or conditions to favor plant growth under stressful situations.

Besides parasitizing other fungi, *T. asperellum* GDFS1009 is capable of parasitizing the moth *Ostrinia furnacalis*, a maize pest, when it ingests the BCA conidia, and when inoculated in maize plants, *T. asperellum* GDFS1009 induces POD, SOD, proline, protease, and PPO activities, increasing defense against the moth, and the co-inoculation with the well-known entomopathogen *Beauveria bassiana* has a better protection effect in the plants [131].

It is noteworthy the potential of *T. asperellum* as a mycoparasite, showing that it cannot only parasitize in a strong manner fungal pathogens but other plant pests such as moths. Moreover, the resistance of this fungus to abiotic stresses makes it a good candidate for agricultural uses in extreme conditions, protecting and promoting the growth of important crops under these circumstances.

#### 4.3.2. Secondary Metabolites

As mentioned before, *vel1* is involved in secondary metabolite biosynthesis. Its overexpression in *T. asperellum* induces the expression of defense-related genes in maize plants, conferring resistance against the pathogens *Cohilohorus herostrophus* and *Fusarium verticilloides* and the co-culture of *T. asperellum* with *B. amyloliquefaciens* provides better protection against the pathogens [143].

Secondary metabolites from *T. asperellum* that can induce defense responses in plants include Epl1-Tas, which induces the expression of genes related to the SA-mediated defense pathway (*NPR1, TGA,* and *PR1*), JA-mediated defense pathway (*COI1*, *JAZ*, *MYC2*, and *ORCA3*) and auxin signaling (*TIR1* and *ARF1*) in *Populus davidiana* × *P. alba* var. *pyramidalis* (PdPap), and increases defense-related enzymatic activity, conferring over 90% more resistance to the pathogen *A. alternata* [137]. The expression in planta of the class II hydrophobin HFBII-4 from *T. asperellum* ACCC30536 in PdPap plants alters the expression of genes related to auxin signaling, SA and JA defense pathways, and defense-related enzymatic activity (PAL, POD, PPO enzymes), reducing ROS accumulation and diminishing lesion area caused by *A. alternata* [138].

Secondary metabolites from *T. asperellum* GDFS1009 contained in fungal fermented broth, alone or in combination with *B. amyloliquefaciens*, showed antagonistic activity against *F. graminearum, F. oxysporum,* and *B. cinerea*, and conferred resistance to *F. graminearum* in wheat plants [139]. Ethyl acetate extract and the fungal filtrate from *T. asperellum* IMI 393,899 showed growth inhibition activity and strong cytotoxic activity against 25 pathogens, including *Penicillium* spp., *Aspergillus* spp., *Fusarium* spp., *Neofusicoccum* spp., *Colletotrichum* spp. and *Phytophthora* spp. [92].

Some of the secondary metabolites from *Trichoderma* species that have antibiotic activity are peptaibols [63], such as crude fungal extract containing peptaibols from *T. asperellum* IRAN 3062C, showing the growth inhibition of *Micrococcus luteus, R. solani* and *A. solani,* and inhibition of spore germination in *A. solani, R. solani* and *Fusarium monilifome* [140]. Another SM, such as the VOC 6-PP and the filtrate from *T. asperellum* P1, inhibits the growth of the maize pathogen *Magnaporthiopsis maydis* [141]. In field conditions, *T. asperellum* P1 confers resistance in maize plants to *M. maydis* [162]. Filtered fermentation extract from *T. asperellum* GDFS1009 inhibited the growth of the pathogens *F. oxysporum* f.sp. *cucumerinum* Owen and *F. graminearum* in 67.59% and 100%, respectively, and induced resistance in tobacco and cucumber plants, observed as increased defense-related enzymatic activity [135]. The crude citric acid extract from *T. asperellum* showed antagonistic capacity against *F. oxysporum* f.sp. *lycopersici*, inhibiting its growth and diminishing the severity of the symptoms caused by this pathogen in tomato plants, increasing PPO and POD enzymatic activity [142].

Secondary metabolites such as VOCs can have growth inhibitory effects over pathogens, such as VOCs released by *T. asperellum* T76-14, which in vitro assays show growth inhibition of the melon pathogen *Fusarium incarnatum*, also preventing the postharvest rot in melon fruits caused by this pathogen [144]. Both VOCs and liquid fermentation extract from *T. asperellum* 6S-2 can inhibit the growth of the pathogen *Fusarium proliferatum* f.sp. *malus domestica* MR5 and the liquid extract also showed the capacity to inhibit pathogen spore formation [133].

The secondary metabolites, both volatile and non-volatile, produced by *T. asperellum* show the capacity to inhibit fungal pathogen growth and to induce plant defense systems. This suggests the versatility of the SM from this fungus to act as an important biocontrol mechanism and its potential to be used in agriculture.

#### 4.3.3. Plant Defense Induction and Priming

*T. asperellum* TaspHu1 increases resistance in tomato plants against *A. alternata* inducing the plant defense pathways, which was observed by an increase in *JAR1, MYC2, NPR1, PR1,* and *GH3.2* gene expression, genes involved in JA- and SA-mediated defense signaling [130]. *T. asperellum* T42 induces a hypersensitive response (HR) in *Pisum sativum* plants upon infection with *Erysiphe pisi*, observed as an increased antioxidant enzymatic activity and lignin accumulation in the plants, and the co-culture with *Pseudomonas fluorescens* has a stronger HR induction in the plants [145]. *T. asperellum* induced priming, increased defense-related enzymatic activity, and antioxidant enzymatic activity in chili pepper plants upon infection with *C. truncatum*, diminishing the symptoms caused by the pathogen and conferring resistance in the plant [112]. Root dipping with *T. asperellum* T1 increases β-1,3-glucanase, chitinase, POX, and phenol oxidase activity in lettuce, inducing resistance against the pathogens *C. cassiicola* and *C. aeria* [134].

The inoculation of *T. asperellum* in tomato plants reduces the ROS accumulation caused by the pathogens *B. cinerea* and *F. oxysporum¸. It* induces ISR in the plants upon *B. cinerea* infection, reducing the symptoms caused by the pathogen [146].

Along with the strong mycoparasitic capacity of *T. asperellum,* its ability to induce plant resistance and confer protection against different pathogens makes this fungus an extraordinary example of an efficient BCA that is already one of the most ubiquitous *Trichoderma* species. Thus, its application in agricultural fields may be facilitated.

### 4.4. Trichoderma Virens

*T. virens* is a ubiquitous fungus isolated from soil and plant matter. In nature, two strains can be identified and distinguished by their secondary metabolite production: strains “Q” and strains “P”. Q strains are characterized by the production of gliotoxin, dimethylgliotoxin, viridiol, and viridin. Meanwhile, P strains produce gliovirin, heptelidic acid, viridiol, and viridin, but no gliotoxin nor dimethylgliotoxin [185].

Gliotoxin and gliovirin are two important metabolites produced by this fungus with strong toxic activity and roles in the establishment of beneficial interactions with plants and in pathogenic interactions with plant pathogens [186,187]. Hence, the strong use of secondary metabolites as a primary mechanism of biocontrol by this fungus. Nonetheless, mycoparasitism is also important for the biocontrol capacity of *T. virens*, along with the induction of plant defense responses to protect plants against different pathogens.

#### 4.4.1. Parasitism and Competition

*T. virens* is an effective mycoparasite and antagonist of several plant fungal pathogens, such as *F. oxysporum* f.sp. *physalia*, whose growth is inhibited in dual confrontation with *T. virens* Gl006, alone or in combination with *Bacillus velezensis* Bs006 supernatant. Nonetheless, the major reduction in the pathogen’s growth is when confronted with the BCA alone (above 70%) [147]. In dual confrontation assays, *T. virens* ZT05 showed mycoparasitic activity over R. solani, penetrating the pathogen hyphae [148], thus inhibiting its growth. Its mycoparasitic abilities have been known for several decades, when it was first observed as coiling hyphae around *R. solani* back in 1932 by Weindling, R. [50].

#### 4.4.2. Secondary Metabolites

Secondary metabolites produced by microorganisms can be tested using cell-free supernatants, such as the cell-free supernatant from *T. virens* Gl006, which alone or in combination with cells or cell-free supernatant from *B. velezensis* Bs006, has antagonistic activity over *F. oxysporum* f.sp. *physalia,* diminishing disease severity caused by this pathogen in *Physalia peruviana* plants [147]. Culture filtrates from *T. virens* TriV_JSB100, or the fungus, induces priming in tomato plants upon infection with *F. oxysporum* f.sp. *lycopersici*, diminishing the symptoms caused by the pathogen [154]. Jogaiah and collaborators [154] also noted that the inoculation of *T. virens* induced the JA-mediated defense pathway in the plant. In contrast, the fungal culture filtrate primarily induces the SA-mediated defense pathway, resulting in an overall resistance to the pathogen.

Some secondary metabolites are antibiotics, such as viridin from *T. virens* IMI 304061, one of the main SMs found in the culture filtrate from an SM-overexpressing strain from *T. virens* IMI 304061, named G2, which has better antibiosis effect over the pathogen *Pythium aphanidermatum* and confers greater protection in *Cicer arietinum* plants against *Sclerotium rolfsii* [149]. Gliotoxin from *T. virens* T23 is important to control *S. rolfsii*, causing structural damage to the pathogen hyphae [188]. The volatile and non-volatile SMs from *T. virens* ZT05 showed growth inhibition of *R. solani* at 80.1% and 63.32% respectively. The non-volatile SMs repressed defense-related enzymatic activity in *R. solani*, indicating that the BCA could regulate the defense mechanism of the pathogen against the mycoparasite [148].

Chitinase and cellulase protein activity are important traits in BCA. Several *T. virens* mutant strains with enhanced chitinase and cellulase activities showed to be more effective at inhibiting *R. solani* growth in dual confrontation assays than the *T. virens* wild-type strain [150].

Secondary metabolites biosynthesis is regulated by different enzymes, such as p450 monooxygenases [189]. *Tvcyt2* is a member of the p450 monooxygenases in *T. virens* and is involved in SM biosynthesis [151]. Ramírez-Valdespino and collaborators [151] found that the overexpression of *tvcyt2* results in a higher concentration of SMs, leading to an increased antagonistic activity against *R. solani* AG2 and a stronger JA- and SA-mediated defense response in *Arabidopsis* plants.

Some other SMs can induce plant resistance, such as the intracellular siderophore ferricrocin from *T. virens*, which is involved in inducing ISR in maize upon infection with the pathogen *Cochliobolus heterostrophus*, since null mutants in the gene *tex10*, the one coding for ferricrocin, failed to induce ISR in the maze, and were more aggressive at colonizing the plants [152]. TvPG2, a constitutive endopolygalacturonase from *T. virens* I10, is involved in inducing ISR in tomato plants against *B. cinerea*, regulating the expression of the inducible *tvpg1* gene coding for TvPG1 endopolygalacturonase, which leads to the resistance against the pathogen [153].

#### 4.4.3. Plant Defense Induction and Priming

*T. virens* can induce plant defense systems, conferring resistance to different pathogens; for example, *T. virens* IARI-P3 induces *PR10* gene expression in susceptible and resistant *Vigna radiata* plants when infected with *R. solani*, reducing significatively disease symptoms caused by the pathogen [155]. *T. virens* induces ISR in maize plants by increasing gene expression of two oxylipins coding genes, 12-OPDA (12-Oxo-10(Z),15(Z)-phytodienoic acid) and an ᵧ-ketol, 9,10-KODA (10-oxo-9-hydroxy- 12(Z), 15(Z)-octadecadienoic acid), granting resistance to the pathogen *Colletotrichum graminicola* [156,157].

*T. virens* has been one of the most used BCA among the *Trichoderma* genus. It is an important secondary metabolite producer with biocontrol activity against many phytopathogens. Its secondary metabolites show great potential to be used in agriculture to control phytopathogens and to induce plant protection against them. Besides, along with other secondary metabolites from *Trichoderma* spp., compounds such as gliotoxin are showing medical applications as possible treatments against cancer [190,191,192], suggesting the span of applications that this fungus has, not being limited to agricultural uses.

### 4.5. Trichoderma Longibrachiatum

*T. longibrachiatum* is frequently isolated from agricultural soils, mushrooms, and marine environments, and it grows better at tropical temperatures rather than in temperate climates [193]. This fungus has been reported to cause cardiac and pulmonary mycoses in immunocompromised humans [194,195,196]. Nonetheless, it has also been reported to be used as an important biocontrol agent [159,169], exerting parasitism and inducing plant defense systems, along with the production of several important secondary metabolites, as shown by the examples described below.

#### 4.5.1. Parasitism and Competition

In dual confrontation assays, *T. longibrachiatum* EF5 showed mycoparasitic activity against *M. phaseolina*, showing hyphal entanglement between both fungi [161], and antagonistic activity with mycelia modifications on *M. phaseolina* and *S. rolfsii* [158]. *T. longibrachiatum* (TG1) coils around *Fusarium pseudograminearum* in a mycoparasitic interaction. In a tripartite interaction with wheat plants under salt stress conditions, the BCA reduces the disease symptoms caused by the pathogen [159]. Tested under field conditions, *T. longibrachiatum* T7407 had a negative effect in the presence of the pathogen *Magnaporthiopsis maydis* in soil by competing with it, thus protecting maize plants from this pathogen and diminishing disease incidence [162]. Besides being a mycoparasite, *T. longibrachiatum* T6 can parasitize eggs and second-stage juveniles from the plant pathogen nematode *Heterodera avenae*, reducing its viability [160].

The parasitic ability of *T. longibrachiatum* is a trait that should be exploited more, especially since it is a parasite of important fungal and nematode phytopathogens, but should be taken carefully under field applications, considering that it is the only fungus from the genus *Trichoderma* to be reported as an opportunistic human pathogen so far.

#### 4.5.2. Secondary Metabolites

Peptaibols are SMs with antibiotic activity, as mentioned before [63]. The crude fungal extract from *T. longibrachiatum* containing peptaibols showed antibacterial activity against the pathogen *M. luteus* [140]. Synthetic analogs to the peptaibol Trichogin GA IV from *T. longibrachiatum* are effective antagonistic compounds to inhibit *Pyricularia oryzae*, a rice pathogen. They can reduce disease symptoms in barley and rice plants [164], which indicates that those synthetic analogs could be used as biocidal compounds instead of chemical compounds. The crude fungal extract containing peptaibols from *T. longibrachiatum* IRAN 3067C showed growth inhibition of several plant pathogens, mainly effective against *R. solani* and *A. solani* [140].

Other SMs with antibiotic activity includes dendrobine from *T. longibrachiatum* MD33, which is an endophyte of the plant *Dendrobium nobile*, known to be the only plant producing dendrobine [163,197]. Sarsaiya and collaborators [163] showed that the *T. longibrachiatum* MD33 has strong antibacterial activity against *Bacillus subtilis, B. mycoides,* and *Staphylococcus* sp., showing the potential of this BCA to inhibit pathogenic bacteria. Three cyclodepsipeptides and six sesquiterpenes compounds identified among the SM produced by *T. longibrachiatum* showed antibiotic activity against several plant pathogens [166]. Only the three cyclodepsipeptides and two sesquiterpenes identified by Du and collaborators [166] inhibited the growth of the nematode *Meloidogyne incognita*, and just the remaining four sesquiterpenes were able to inhibit the fungal pathogens *Colletotrichum lagrnarium*, *C. fragariae, B. cinerea* PTQ1, and CMQ1, *F. oxysporum* f.sp. *cucumerinum* and *F. oxysporum* f.sp. *lycopersici*, showing pathogen specificity of the SM tested. Culture filtrate containing 13 SMs, known as sorbicillinoids, from *T. longibrachiatum* SFC100166 showed in vitro growth inhibition of the pathogens *Alternaria brassicola*, *B. cinerea, Colletotrichum coccodes*, *Cladosporium cucumerinum*, *Cylindrocarpon destructans*, *Magnaporthe oryzae* and *Phytophthora in festans* [167]. When tested separately, eleven of the sorbicillinois identified inhibited the pathogens tested, with *P. infestants* being the most affected by the compounds, and four sorbicillinois were able to induce resistance in tomato plants against this pathogen [167]. VOCs from *T. longibrachiatum* EF5 inhibited the growth of the pathogens *S. rolfsii* (57%) and *M. phaseolina* (35%) by altering mycelia structure [158], suggesting that these compounds may be important to the biocontrol traits of *T. longibrachiatum*.

Some secondary metabolites induce plant defense responses, such as the hydrophobin HYTLO1 from *T. longibrachiatum* MK1, which is perceived by *Lotus japonicus*, activating the expression of Ca^2+^-mediated signaling, leading to the induction of defense-related genes in the plant [165].

Some SMs from *T. longibrachiatum* have biocontrol activity over other organisms, such as the ethyl acetate extract from *T. longibrachiatum* AUMC 5125 that has effective antibiotic activity over the cotton aphid *Aphis gossypii* [168]. Fermentation crude extract from *T. longibrachiatum* T6 showed antagonistic activity against eleven phytopathogens tested, being especially effective against *Valsa mali¸* inhibiting up to 95% [169].

As shown by the examples above, secondary metabolites from *T. longibrachiatum* play important roles in its biocontrol capacity, and considering that this fungus may cause human diseases, SMs could be studied to be used alone, without the need for the microorganism, diminishing health concerns about the use and introduction of microorganisms in the environment.

#### 4.5.3. Plant Defense Induction and Priming

*T. longibrachiatum* H9, a novel strain, colonized cucumber roots promoting plant growth and inducing JA/Et and SA defense signaling pathways, conferring resistance in cucumber to the pathogen *B. cinerea* [77]. In a greenhouse experiment, *T. longibrachiatum* T6 induced flavonoid and lignin content, as well as defense-related enzymatic activity in wheat roots, conferring resistance against the nematode *H. avenae* [160].

*T. longibrachiatum* has shown to be a versatile biocontrol agent, parasitizing not only fungi but nematodes as well, and it is a good secondary metabolite producer, being used to obtain important compounds such as peptaibols. This fungus has good potential to be an efficient BCA, and its traits could be exploited in field conditions, considering the potential to use SMs in substitution of synthetic compounds.

### 4.6. Trichoderma Viride

*T. viride* has optimal growth at 25 °C and does not grow at 35 °C; it can be isolated from soil and organic matter, some of its strains have a faint coconut smell, and conidia can be observed after 2 days. *T. viride* is considered the type species of the genus *Trichoderma* [173,198], and it is one of the most common species found in soil.

*T. viride* has been used as a biocontrol agent, especially due to its mycoparasitic ability. It has been reported that *T. viride* can mycoparasite fungal pathogens such as *F. moniliforme, Cryphonectria parasitica,* and *Schizophyllum commune* [199,200,201,202]. The use of commercial chitinases derived from *T. viride* causes damage to the silkworm *Bombix mori* [203], suggesting the ability of this fungus to degrade chitin from insects that could cause plant diseases. Below, we present recent examples regarding the biocontrol mechanisms of *T. viride*.

#### 4.6.1. Parasitism and Competition

By competing for space and nutrients in dual culture assays, *T. viride* showed antagonistic activity against the pathogen *Sclerotinia sclerotiorum*, presenting a clear zone of inhibition in the culture plates on day 4 of interaction, indicating antibiosis mechanisms exerted by *T. viride* over the pathogen, and a 67.284% growth inhibition by day 6 of confrontation [170].

In dual confrontation assays, *T. viride* showed antagonistic activity against the pathogens *Fusarium solani*, *R. solani,* and *S. rolfsii*, limiting their growth by 29.76%, 15.27%, and 19.73%, respectively [171].

#### 4.6.2. Secondary Metabolites

*T. viride* produces SM with antifungal activity. The crude mycelial extract and the ethanolic extract from this fungus showed antifungal activity against *Candida albicans*, *Fusarium solani*, *F. oxysporum*, *R. solani*, and *Pythium ultimum*, and antibacterial activity against *Bacillus subtilis*, *Escherichia coli*, and *Pseudomonas fluorescens* showing inhibition zones in the culture plates. In contrast, the VOCs produced by *T. viride* showed antibacterial activity against *B. subtilis* and *E. coli* and antifungal activity against *C. albicans*, *F. solani*, and *R. solani* [171].

VOCs from *T. viride* BHU-V2 showed antagonistic activity against *S. rolfsii*, inhibiting the pathogen growth both in vitro and in soil experiments. It was determined that VOCs are capable of altering the structure of the pathogen hypha, thus limiting its growth [172]. Singh and collaborators [172] also showed that VOCs from *T. viride* increase PAL, PPO, chitinases, and β-1,3-glucanase activity in okra plants, inducing resistance and diminishing cell death caused by *S. rolfsii*.

#### 4.6.3. Plant Defense Induction and Priming

In glasshouse experiments, inoculation of *T. viride* alone or in combination with *Trichoderma erinaceum* suppressed the disease caused by *Sclerotinia sclerotiorum* in *Phaseolus vulgaris* cv. *Anupama* plants. Nonetheless, the combination of the BCAs had better results [170]. Kumar and collaborators [170] also found that plants pretreated with either *Trichoderma* species or their combination reduced ROS accumulation induced by the pathogen, enhancing antioxidant activity in the plants.

Despite being the species reviewed here with less recent literature regarding its biocontrol traits, *T. viride* has been studied as a plant growth promoter or enhancer of desirable traits in plants [204,205] or as an important organism in bioremediation or preparation of surfaces for bioremediation of toxic organic compounds such as toluene [206] or TNT [207] or heavy metals such as lead [208]. These make an interesting *Trichoderma* species to study further in various possible applications, not limited to agricultural uses.

### 4.7. Other Trichoderma Species

Besides being the most used *Trichoderma* species as biocontrol agents and/or plant growth promoters [55], other species are surfacing. *T. lignorum,* in combination with *B. bassiana,* was effective at controlling leafcutter ants (*Atta cephalotes*) populations [209]. *T. koningii* reduced disease severity in grapevines caused by the pathogens *Phaeomoniella chlamydospora* and *P. minimum* [210]. *T. erinaceum* can suppress an infection caused by *S. sclerotiorum* in bean plants [170]. It is also capable of overgrowing *R. solani* and inducing ROS and defense-related enzymatic activity in rice plants [211]. *T. citrinoviride* resulted in an effective mycoparasite of six pathogens of ginseng plants: *R. solani*, *B. cinerea, Alternaria panax*, *Cylindrocarpon destructans*, *Phytophthora cactorum*, and *Pythium* spp. [212].

These studies about other *Trichoderma* species, and the constant reports of new isolates and their applications in industry and medicine, for example, show the diversity of species belonging to this genus and their potential to be used outside of the traditional plant growth promotion and biocontrol abilities attributed to *Trichoderma* since it was first described. Hence, not only new isolates or species should be of interest, but additional information about the “classic” *Trichoderma* species and its applicability in other fields could be addressed.

## 5. Other Biocontrol Strategies Involving *Trichoderma* spp.

Besides the direct or indirect use of *Trichoderma spp.* in the biocontrol of plant pathogens, that is, applied in field or greenhouse conditions as conidia, hyphae, a mix of its metabolites, other new and forthcoming biocontrol strategies have been arising over the past years. These include the combined use or co-inoculation of *Trichoderma* species with plant growth-promoting bacteria or mycorrhizae, where the co-inoculation of the BCA *T. viride* and several arbuscular mycorrhizal fungi (AMF) such as *Rhizoglomus clarum*, *Funneliformis monosporum*, *Acaulospora laevis*, and *Dentiscutata nigra*, had a positive and synergistic effect on the overall health of *Allium cepa* plants [205], and the co-inoculation of *T. harzianum* with *Bacillus sp.* proved effective in controlling the disease caused by *F. oxysporum* f.sp. *capae* on shallot plants [213].

*Trichoderma* species are not only capable of cooperating with other beneficial microbes, but they can even change the plant rhizosphere microbiome and microbial communities, favoring plant resistance against several pathogens. *T. harzianum* CCTCC-RW0024 modifies the rhizosphere microbiome in maize plants, conferring resistance against *F. graminearum* [73]. Moreover, *T. harzianum* changes microbial communities in the rhizosphere of *Piper nigrum* plants, favoring the presence of other beneficial microbes [214] and diminishing the incidence of *Plasmodiophora brassicae, Alternaria* sp. and *Fusarium* sp. pathogens in the rhizosphere of cabbage plants [215]. The presence of *T. asperellum* M45a in the watermelon rhizosphere modifies microbial composition, increasing the presence of plant-growth-promoting rhizobacteria and reducing the presence of plant pathogenic fungi, and disease incidence caused by *Fusarium oxysporum* f.sp. *niveum* in watermelon plants [216]. *T. asperellum* also modifies endophytic microorganisms’ population in maize stalk, increasing resistance against *F. graminearum* and *F. verticilloides* [217]. This information suggests that changes in the microbiome could have a biocontrol effect on important plant pathogens, thanks to the presence of *Trichoderma* species.

*Trichoderma* has also been used for the green biosynthesis of nanoparticles and their subsequent use in agriculture. Ag, ZnO, and CuO nanoparticles have been biosynthesized using *T. harzianum*, and those nanoparticles showed an inhibitory effect over *A. alternata, Pyricularia oryzae*, and *S. sclerotiorum* [218], and ZnO nanoparticles showed biocontrol capability against *Fusarium* sp., *R. solani* and *M. phaseolina*, three important pathogens of cotton plants [219]. *T. viride* has also been used to synthesize TiO_2_, showing larvicidal and pupicidal effects on *Helicoverpa armigera,* a pest of important crops such as maize, wheat, and beans [220]. This shows that *Trichoderma* can be used as a bio-tool to obtain chemical products that are beneficial in agriculture, in substitution of chemical fungicides [221].

These new approaches aim to minimize the harmful effects on health and the environment that chemical fungicides present. *Trichoderma* seems to have an important role in replacing chemical agricultural products.

## 6. *Trichoderma* Bioformulations in Agriculture for Use in Biocontrol

The use of *Trichoderma* as a biocontrol and plant growth promoter in agriculture is not new. There is significant research about the bioformulations that are most effective in the field, as well as the issues regarding the use and distribution of such products, such as acceptance from the farmers’ community, the introduction of different species in the environment, and their efficacy compared to chemical fertilizers and pesticides [12,222,223,224]. Hence, the importance of producing or manufacturing *Trichoderma*-based products, alone or in combination with other BCA, is their efficacy when tested so that the consumers will receive them well and would be willing to switch to bioformulations and use fewer chemical products on their crops.

Several *Trichoderma-*based formulations have been tested under greenhouse or field conditions, showing positive results such as increased plant growth, production, and resistance against diseases.

Wong and collaborators [62] combined *T. harzianum* CBF2- with *Pseudomonas aeruginosa* DRB1 using four different formulations: Pesta granules, Talc powder, alginate beds, and liquid formulation, and these bioformulations were tested to biocontrol *F. oxysporum* f.sp. *cubense*, the causing agent of banana wilt. Pesta granules and Talc powder were more efficient at diminishing disease symptoms in the plant (66.67% and 58.33%, respectively), followed by alginate beds (46.75%) and liquid formulations (43.06%). The four formulations were better than the application of Benomyl, a known chemical antifungal agent, with only a 37.50% reduction in disease symptoms [62]. As Pesta granules formulation, both BCA were viable for at least 180 days when stored at 4 °C, and the formulation showed better performance in BCA viability and storage [62].

Using five different agro-based wastes (vermicompost, vegetable wastes, used tea leaves, sugarcane bagasse, and cow dung) to grow *T. lixii* TvR1 and use as bio-products, sugarcane bagasse was the most efficient substrate to grow *Trichoderma*, and in pot experiments using spinach, promoted the plant growth [225]. Using a formulation of *T. asperellum* in coconut fiber promoted the growth of tomato plants and conferred resistance against *F. oxusporum* f.sp. *lycopersici* in field trials [226]. *T. harzianum* grown on a spent mushroom substrate (SMS) of *Pleurotus ostreatus* showed growth promotion in tomato plants and increased disease resistance against *F. oxysporum* f.sp. *lycopersici*. The bioformulation used was the best among several substrates, including combinations of SMS with paddy straw [227]. Two formulations from *T. citrinoviride*, dustable powder and granules, were effective against *B. cinerea* and *C. destructans* in vitro. Both were effective at preventing disease caused by *A. panax* in ginseng plants [213].

Seeds of chickpea and lentils treated with a formulation made with the mutant strain *T. virens* G2 on tamarind seeds and talcum powder, named TrichoBARC, improved yield and reduced seed mortality in chickpea and lentils in field trials, and induced resistance against *S. rolfsii* in chickpea plants [149]. Seed-coating with a bioformulation made with *T. harzianum* and chitosan-PEG as the delivery system showed antagonistic activity against *F. oxysporum, M. phaseolina,* and *Aspergillus niger*, the bioformulation also promoted the growth of safflower and groundnut plants, and resistance against *M. phaseolina* and *A. niger* [228].

Commercial formulations T34 Biocontrol (Biocontrol Technologies S.L) from *T. asperellum* T34 and Trianum P (Koppert) from *T. harzianum* T22 induced systemic resistance in tomato plants, repressed reproduction of the plant nematode *Meloidogyne incognita* and, T34 Biocontrol also reduced the nematode infectivity [229]. The commercial product Xedavir made with *T. asperellum* (Xeda International^®^) was tested in vitro against *F. graminearum* and *F. verticilloides* [230]. As spore suspension, Xedavir inhibits germination of *F. graminearum* up to 53%, *F. verticilloides* up to 22%, and as the cell-free extract, Xedavir inhibits *F. graminearum* and *F. verticilloides* germination up to 82% and 76%, respectively. Xedavir also showed the capacity to inhibit the production of the mycotoxin deoxynivalenol (DON) from *F. graminearum* [230].

One concern about the use of BCA in the field is precisely the introduction of species in the environment, which is why some studies are focusing on using local BCA strains [231]. Nonetheless, another option to avoid the use of whole microorganisms is the use of elicitor agents. Nandini and collaborators [232] formulated nanoemulsions using total lipids extracted from six *Trichoderma* species. The nanoemulsion from *T. brevicompactum* showed a remarkable capacity to induce resistance and hypersensitive response in pearl millet plants against the downy mildew pathogen *Sclerospora graminicola*, both in vitro and in field conditions [232], showing the feasibility of using elicitors from the microorganisms, without the necessity for the living organism.

The results of field or greenhouse testing of the *Trichoderma-*based formulations are promising, and studies regarding this issue should complement in vitro assays of its biocontrol capacity, to facilitate the application and distribution of bioformulations.

## 7. Conclusions

The use of chemical pesticides and fertilizers has been detrimental to human and environmental health. That is why the search for more sustainable and environmentally friendly solutions has led to the research of organisms as biocontrol agents. Such as *Trichoderma*, which possesses different biocontrol traits, which makes it one of the most effective organisms studied against various types of plant pathogens, not being limited to controlling fungi and oomycetes, but insects, pests, and nematodes as well, either by limiting their growth by competition, antibiosis, or parasitism, or by enhancing plant protection against them, making this fungus a versatile option to control several phytopathogens.

*Trichoderma* has been used in different types of formulations in agriculture, mainly to promote plant growth and increase crop yield. Nonetheless, the use of *Trichoderma-*based formulations for the control of pathogens also needs to be considered in studying such products, especially under field conditions, since most of the studies that consider this aspect are done in vitro in dual confrontations.

Another interesting point to remark on is the use of secondary metabolites from *Trichoderma* or green biosynthesis of nanoparticles using this fungus, which can be used in agriculture to promote plant growth or to inhibit pathogen growth without the fungus per se, or using *Trichoderma* strains isolated from local environments, eliminating the introduction of foreign strains into the environment.

It is clear that the different *Trichoderma* species are used as mycoparasites, and specific species such as *T. atroviride* or *T. harzianum* are among the strongest and classic mycoparasites. Nonetheless, there are emerging *Trichoderma* species that have been isolated and applied from local areas and are promising candidates as biocontrol agents. *Trichoderma* as biocontrol agents started being studied as mycoparasites. Nonetheless, its use against other plant pathogens such as nematodes and insects is gaining notice due to the different mechanisms it has to exert control of such a variety of plant pathogens, regulating both soil and aerial-borne diseases.

There is still much to be done regarding applying *Trichoderma*-based formulations in field conditions and interaction with other soilborne microorganisms to understand better its interaction within the plant microbiome and its biocontrol traits. This a field to be exploited in depth for further research.

## Figures and Tables

**Table 1:** Main *Trichoderma* species used in agriculture and their biocontrol traits.

Trichoderma Species	Biocontrol Traits	Biocontrol Effect	Reference
*T. atroviride*	Parasitism and competition	Competition and mycoparasitism inhibit the growth of several plant pathogens	[92]
		Competition against *Ph. cinnamomi* inhibits the pathogen growth	[93]
		Competition inhibits the growth of *N. parvum*	[94]
		Competition and antagonistic activity against *F. avenaceum* and *F. culmorum*	[95]
	Secondary metabolites production and antibiosis	Ethyl acetate extract inhibits growth and has antifungal activity against 25 plant pathogens	[92]
		Swollenin TaSwo1 confers protection in *Capsicum annum* plants against *A. solani* and *R. solani*	[96]
		Vel1-derived secondary metabolites and parasitism-related enzymatic activity influences mycoparasitic activity against *F. graminearum*	[97]
		Fungal culture inhibits the growth of pathogen *F. avenaceum*	[95]
		Fermented culture inhibits the growth of *B. cinerea*	[98]
		Tal6, a LysM effector, antagonizes several plant pathogens	[99]
		VOCs inhibit the growth of pathogen *F. avenaceum*	[95]
		6-PP production under dark conditions enhances antagonistic activity against *R. solani* and *F. oxysporum*	[100]
		VOCs inhibit the growth of *R. solani*, *B. cinerea,* and *F. oxysporum*, conferring resistance in *Arabidopsis* plants	[101]
	Plant defense induction/Priming	SA induced defense response in grapevine Tempranillo cultivar, protecting the plant against *N. parvum*	[94]
		Increasing the defense-related enzymatic activity in tomato plants confers resistance against *B. cinerea* and diminishes the disease’s symptoms	[98]
		Modification of gene transcripts related to plant defense, and induction of plant-defense VOCs, confer resistance to the moth *S. littotalis* and the aphid *M. euphorbiae* in tomato plants	[102]
		Priming JA and SA defense pathways increased gene expression confers resistance against *B. cinerea* in *Arabidopsis* plants	[103]
*T. harzianum*	Parasitism and competition	Growth inhibition of *F. oxysporum* in in vitro confrontations	[104]
		Competition for nutrient and space and mycoparasitism inhibits the growth of *F. sudanense*	[105]
		Growth inhibition of *A. cerealis* in in vitro confrontations	[106]
		Mycoparasitism inhibits the growth of *F. oxysporum*	[107]
		Growth inhibition of several postharvest pathogens of sweet potato in in vitro assays	[108]
		Mycoparasitism inhibits the growth of *F. oxysporum*, *A. alternata*, *A. flavus*, and *A. carbonarius*	[109]
		Competition for space with the pathogen *F. pseudograminearum* in the rhizosphere soil of wheat plants	[110]
		Mycoparasitism of *F. graminearum* inhibits the pathogen growth	[111]
		Growth inhibition of *C. truncatum* in in vitro confrontations	[112]
	Secondary metabolites production and antibiosis	6-PP application on maize roots diminishes root damage by the scarab *P. vetula*	[113]
		Secondary metabolite production regulated by the transcriptional coactivator ThMBF-1 is important to inhibit the growth of *B. cinerea* and *F. oxysporum* and to confer resistance in tomato plants	[114]
		Reduction of cucumber mosaic virus infection on cowpea plants by three peptaibols: trichorzins HA II, HA V, and HA VI.	[115]
		Aspartic protease P6281 inhibits the growth and spore formation of *B. cinerea*, *M. circinelloides*, *A. flavus*, *A. fumigatus* and inhibits the growth of *R. solani*	[116]
		Chitinase activity and hydrophobicity are essential for the mycoparasitism of *R. solani*	[117]
		SMs from the fungal extract inhibit the growth of *F. graminearum*	[73]
		Culture filtrate from *T. harzianum* has antifungal activity against *F. oxysporum*	[107]
		The enzyme ThLAAO induces the expression of defense-related genes in tobacco plants, conferring resistance against *B. cinerea* and *S. sclerotiorum*	[118]
		Epl-1 down-regulates virulence genes in *B. cinerea* during in vitro confrontations	[119]
		Culture filtrate from *T. harzianum* inhibits *P. ultimum* growth	[120]
		SM from culture filtrates reduce the growth of several plant pathogens	[121]
		Metabolites extracts alleviate the symptoms caused in wheat seedlings by the pathogen *F. culmorum*	[122]
		In planta expression of ThKEL1 induces the expression of genes involved in SA and JA pathways in *Arabidopsis* and rapeseed plants, conferring resistance against *B. cinerea*	[123]
	Plant defense induction/Priming	JA signaling induction in tomato against the feeding insect *N. viridula*	[124]
		SA and JA/Et signaling induction in tomato plants upon infection with the nematode *M. incognita*	[125]
		Strong VOC priming in tomato plants to attract the parasitoid *A. ervi* to exert biocontrol over the aphid *M. euphorbiae*	[126]
		Induction of antioxidant enzymes in tomato plants upon *F. oxysporum* infection	[104]
		Induction of Et, JA, ISR pathways, and isoprenoid biosynthesis in tomato plants upon *M. euphorbiae* infestation	[127]
		Induction of defense-related enzymes, SA accumulation, and phenolic compounds in wheat, conferring resistance to *B. sorokiniana*	[128]
		Induction of several plant defense-related compounds in tomato plants upon infection with *A. cerealis*	[106]
		Increased protection and reduction of cell death in soybean plants upon *F. oxysporum* infection	[107]
		SA signaling pathway and priming are induced by Epl-1 in tomato plants against *B. cinerea*	[119]
		Induction of antioxidant activity and redox homeostasis in cucumber plants promotes resistance against *F. oxysporum*	[129]
		Induction of priming, defense-related enzymatic activity, antioxidant enzymatic activity, and reduction of ROS accumulation in chili pepper plants, protecting and reducing symptoms from *C. truncatum* disease	[112]
*T. asperellum*	Parasitism and competition	Competition and mycoparasitism inhibit the growth of eight phytopathogens	[130]
		Parasitism of the maize moth pathogen *O. furnicalis*, inducing enzymatic activity related to plant defense in maize	[131]
		Growth inhibition of *C. truncatum* in in vitro confrontations	[112]
		Competition and mycoparasitism inhibit the growth of several plant pathogens	[92]
		Growth inhibition of *R. solani* and *A. alternata* under salt stress conditions	[132]
		Competition and mycoparasitism inhibit the growth of *F. proliferatum* f.sp. *malus domestica* MR5, and other plant pathogens	[133]
		Growth inhibition of the lettuce pathogens *C. cassiicola* and *C. aeria*	[134]
		Competition and mycoparasitism inhibit the growth of *F. oxysporum* f.sp. *cucumerinum* Owen and *F. graminearum*	[135]
		Mycoparasitism inhibits the growth of *P. noxius* and confers resistance in *E. japonica* plants	[136]
	Secondary metabolites production and antibiosis	Elicitor protein Epl1-Tas induces enzymatic activity related to plant defense response in *P. davidiana* × *P. alba* var. *pyramidalis*, conferring resistance against *A. alternata*	[137]
		Hydrophobin HFBII-4 induces enzymatic activity and gene expression related to plant defense response in *P. davidiana* × *P. alba* var. *pyramidalis*, conferring resistance against *A. alternata*	[138]
		The fermented broth has antifungal activity against *F. oxysporum, F. graminearum*, and *B. cinerea* and increases the resistance of wheat against *F. graminearum*	[139]
		The crude extract containing peptaibols inhibits spore germination of *A. solani, R. solani,* and *F. moniliforme*, and it has antibacterial activity against *M. luteus*	[140]
		Crude extract and 6-PP inhibit the growth of *M. maydis*	[141]
		Ethyl acetate extract inhibits growth and has antifungal activity against 25 plant pathogens	[92]
		Liquid fermentation extract inhibits *F. proliferatum* f.sp. *malus domestica* MR5 growth and spore germination	[133]
		Filtered fermentation liquor inhibits *F. graminearum* growth	[135]
		Crude citric extract inhibits *F. oxysporum* f.sp. *lycopersici* growth and induces enzymatic activity related to plant defense response in tomato plants	[142]
		Vel1-derived SM induces the expression of defense-related genes in maize plants, conferring resistance against *C. herostrophus* and *F. verticilloides*	[143]
		VOCs prevent postharvest rot caused by *F. incarnatum* in *Cucumis melo* fruits, and they inhibit pathogen growth	[144]
		VOCs inhibit *F. proliferatum* f.sp. *malus domestica* MR5 growth	[133]
	Plant defense induction/Priming	Induction of defense-related genes in tomato plants, granting resistance against *A. alternata*	[130]
		Induction of hypersensitive response in *Pisum sativum* plants in response to the pathogen *E. pisi*	[145]
		Induction of systemic resistance and reduction of ROS accumulation in tomato leaves upon infection with *F. oxysporum* and *B. cinerea*	[146]
		Induction of priming, defense-related enzymatic activity, antioxidant enzymatic activity, and reduction of ROS accumulation in chili pepper plants, protecting and reducing symptoms from *C. truncatum* disease	[112]
		Induction of defense-related enzymatic activity in lettuce plants upon infection with *C. cassiicola* and *C. aeria*	[134]
*T. virens*	Parasitism and competition	Antagonistic and mycoparasitic activity against *F. oxysporum* f.sp. *physalia*, diminishing disease severity in *Physalis peruviana* plants	[147]
		Mycoparasitic activity against *R. solani*.	[148]
	Secondary metabolites production and antibiosis	Excess production of secondary metabolites enhances antibiosis and mycoparasitic capacity against *P. aphanidermatum* and *S. rolfsii* and confers protection on *Cicer arietinum* plants against *S. rolfsii*	[149]
		Chitinase and cellulase activity inhibit *R. solani* growth	[150]
		Secondary metabolites inhibit the growth of *R. solani* AG2 and induce JA and SA accumulation in *A. thaliana* plants	[151]
		Non-volatile secondary metabolites inhibit the growth of *R. solani* and downregulate genes coding for defense enzymatic activity in the pathogen	[148]
		Ferricrocin, a siderophore, is involved in ISR induction in maize against *C. heterostrophus*	[152]
		Endopolygalacturonase TvPG2 induces resistance in tomato plants against *B. cinerea* via ISR induction	[153]
		Cell-free supernatant inhibits the growth of *F. oxysporum* f.sp. *physalia*, and confers resistance in *P. peruviana* plants	[147]
		Culture filtrate induces ISR in tomato plants, conferring resistance to *F. oxysporum* f.sp. *lycopersici*. Priming and induction of JA defense pathway in tomato plants against *F. oxysporum* f.sp. *lycopersici*	[154]
		Volatile secondary metabolites inhibit *R. solani* growth	[148]
	Plant defense induction/Priming	Induction of defense-related genes confers resistance against *R. solani* in *Vigna radiata* susceptible and resistant varieties.	[155]
		Induced systemic resistance in maize plant against *C. graminicola*, via the induction of oxylipins and ketol, as ISR signals	[156,157]
*T. longibrachiatum*	Parasitism and competition	Competition and mycoparasitism inhibit the growth of six phytopathogens, being more effective against *R. solani* and *A. solani*	[140]
		Competition and antibiosis inhibit the growth of *Sclerotium rolfsii* and *M. phaseolina*	[158]
		Mycoparasitism inhibits the growth of *F. pseudograminearum*	[159]
		Parasitism of eggs and second-stage juveniles of *H. avenae*	[160]
		Mycoparasitism inhibits the growth of *M. phaseolina*	[161]
		Competition diminishes the presence of *Magnaporthiopsis maydis* in maize plants and its negative effect on plant growth and disease symptoms in field conditions	[162]
	Secondary metabolites production and antibiosis	The crude extract containing peptaibols has antibacterial activity against *M. luteus*	[140]
		Dendrobine has antibacterial properties against plant-pathogenic bacteria	[163]
		Synthetic analogs to the peptaibol Trichogin inhibit the growth of *Pyricularia oryzae*, reduce disease symptoms in rice and barley plants, and alter the spore and mycelial structure of the pathogen	[164]
		The hydrophobin HYTLO1 induces the expression of defense-related genes in *Lotus japonicus* plants	[165]
		Metabolites inhibit the growth of *M. phaseolina*	[161]
		Sesquiterpenes and cyclodepsipeptides inhibit the growth of several plant fungal pathogens and the nematode pathogen *M. incognita*	[166]
		Culture filtrate and sorbicillinoids inhibit the growth of several plant pathogens and confer resistance in tomato plants against *Ph. infestans*	[167]
		Ethyl acetate extract has effective toxicity against the cotton aphid *A. gossypii*	[168]
		Fermentation crude extract and fungicide compounds inhibit the growth of the pathogen *V. mali*	[169]
		VOCs inhibit the growth of *S. rolfsii* and *M. phaseolina*	[158]
	Plant defense induction/Priming	Induction of JA/Et and SA pathways, conferring resistance in cucumber plants against *B. cinerea*	[77]
		Induction of defense-related enzymatic activity and flavonoids and lignin content in wheat roots upon infection with *H. avenae*	[160]
*T. viride*	Parasitism and competition	Competition inhibits *S. sclerotiorum* growth in dual confrontations	[170]
		Competition inhibits *F. solani*, *R. solani*, and *S. rolfsii* growth in dual confrontations	[171]
	Secondary metabolites production and antibiosis	VOCs show antibacterial and antifungal activity	[171]
		VOCs inhibit the growth of *S. rolfsii* in soil and in dual confrontations, affecting the mycelial structure. VOCs induce defense-related enzymatic activity in okra plants upon infection with *S. rolfsii*	[172]
		Crude and ethanol extract show antibacterial and antifungal activity	[171]
	Plant defense induction/Priming	Induction of antioxidant enzymatic activity and reduction of ROS accumulation in *Phaseolus vulgaris* plants upon infection with *S. sclerotiorum*	[170]

## Data Availability

Not applicable.
